# Life on the edge: O_2_ binding in Atlantic cod red blood cells near their southern distribution limit is not sensitive to temperature or haemoglobin genotype

**DOI:** 10.1242/jeb.141044

**Published:** 2017-02-01

**Authors:** Samantha L. Barlow, Julian Metcalfe, David A. Righton, Michael Berenbrink

**Affiliations:** 1 Department of Evolution, Ecology and Behaviour, Institute of Integrative Biology, The University of Liverpool, Biosciences Building, Crown Street, Liverpool L69 7ZB, UK; 2 Centre for Environment, Fisheries and Aquaculture Science (CEFAS), Lowestoft NR33 0HT, UK

**Keywords:** Climate change, *Gadus morhua*, Oxygen transport, O_2_ affinity, Thermal tolerance, Bohr effect

## Abstract

Atlantic cod are a commercially important species believed to be threatened by warming seas near their southern, equatorward upper thermal edge of distribution. Limitations to circulatory O_2_ transport, in particular cardiac output, and the geographic distribution of functionally different haemoglobin (Hb) genotypes have separately been suggested to play a role in setting thermal tolerance in this species. The present study assessed the thermal sensitivity of O_2_ binding in Atlantic cod red blood cells with different Hb genotypes near their upper thermal distribution limit and modelled its consequences for the arterio-venous O_2_ saturation difference, *S*a–v_O_2__, another major determinant of circulatory O_2_ supply rate. The results showed statistically indistinguishable red blood cell O_2_ binding between the three HbI genotypes in wild-caught Atlantic cod from the Irish Sea (53° N). Red blood cells had an unusually low O_2_ affinity, with reduced or even reversed thermal sensitivity between pH 7.4 and 7.9, and 5.0 and 20.0°C. This was paired with strongly pH-dependent affinity and cooperativity of red blood cell O_2_ binding (Bohr and Root effects). Modelling of *S*a–v_O_2__ at physiological pH, temperature and O_2_ partial pressures revealed a substantial capacity for increases in *S*a–v_O_2__ to meet rising tissue O_2_ demands at 5.0 and 12.5°C, but not at 20°C. Furthermore, there was no evidence for an increase of maximal *S*a–v_O_2__ with temperature. It is suggested that Atlantic cod at such high temperatures may solely depend on increases in cardiac output and blood O_2_ capacity, or thermal acclimatisation of metabolic rate, for matching circulatory O_2_ supply to tissue demand.

## Abstract (precis)

**Highlighted Article:** Red blood cell oxygen binding affinity in Atlantic cod near their southern, warmer limit of distribution is largely temperature independent and not affected by functional differences between their major haemoglobin genotypes.

## INTRODUCTION

The 5th assessment report of the Intergovernmental Panel on Climate Change documents an increase in average global sea surface temperatures over the last century and predicts their continued rise (IPCC, 2014). The body temperature of marine ectothermic organisms is directly affected by warming seas, which makes an understanding of their physiological capabilities to withstand elevated temperatures vital for predicting future redistributions of species and influencing management regimes (e.g. Deutsch et al., 2015).

Atlantic cod (*Gadus morhua*) are widely distributed in coastal and shelf seas throughout the North Atlantic, but stocks near the southern, equatorward upper thermal margin of their historic distribution limit in the Irish and southern North Sea have declined over the past decades, which has in part been ascribed to warming seas (Brander, 2005; Drinkwater, 2005; Perry et al., 2005; Beggs et al., 2014; Deutsch et al., 2015). Given, in addition, the high commercial importance of cod and the resulting fishing pressures, this has led to extensive research into thermal effects on Atlantic cod life history traits, physiology, behaviour, abundance and distribution (Mork et al., 1984; Petersen and Steffensen, 2003; Gamperl et al., 2009; Righton et al., 2010; Behrens et al., 2012; Engelhard et al., 2014; Kreiss et al., 2015; Rutterford et al., 2015). Based on the thermal sensitivity of life history traits and projected future temperature changes, Atlantic cod stocks near their current upper thermal distribution limit in the north-east Atlantic have been predicted to disappear entirely from the Celtic and Irish Seas by the end of this century (Drinkwater, 2005). Likewise, alternative mechanistic models based on a metabolic index of the O_2_ supply to demand ratio and projected future temperature and O_2_ partial pressure (*P*_O_2__) changes predict reductions in the current habitat volume (occupied area×depth range) by 12–32% at the equatorward upper thermal margin of Atlantic cod by the end of the present century (Deutsch et al., 2015).

The oxygen- and capacity-limited thermal tolerance (OCLTT) hypothesis attempts to provide a general mechanistic explanation for the thermal distribution limits of aquatic organisms, suggesting that the capacity of O_2_ supply mechanisms in aquatic ectotherms, such as the circulatory and ventilatory systems, becomes insufficient to meet rising O_2_ demands at thermal extremes, thus affecting their ability to maintain an adequate aerobic scope for activities such as feeding, digestion, growth, migration, reproduction and predator evasion (Pörtner, 2001; Pörtner and Knust, 2007).

Studies on the acute thermal tolerance of Atlantic cod have identified the circulatory system as a primary limiting factor in the O_2_ supply cascade from the environment to the tissues, with cardiac function suggested to become compromised close to the critical thermal maximum (Sartoris et al., 2003; Lannig et al*.*, 2004; Gollock et al., 2006). According to the Fick equation, cardiac output, *Q̇* (the product of heart rate, *f*_H_, and stroke volume, *V*_S_) and the arterio-venous O_2_ difference, *C*a_O_2__−*C*v_O_2__, together determine the rate of circulatory O_2_ delivery (*Ṁ*_O_2__) between respiratory organs and tissues (Fick, 1870):
(1)
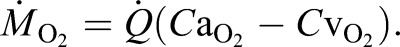


The contribution of changes in *C*a_O_2__−*C*v_O_2__ in the assessment of maximal O_2_ supply capacities during warming of marine ectotherms is largely unknown, although it has long been recognised that in humans, for example, the increase in *C*a_O_2__−*C*v_O_2__ may surpass the increase in *Q̇* in its contribution to meeting elevated *Ṁ*_O_2__ during heavy exercise (factorial increases of 3.45 and 2.51, respectively; Ekelund and Holmgren, 1964; Dejours, 1975). *C*a_O_2__−*C*v_O_2__ essentially equals the maximal blood O_2_ binding capacity multiplied by the arterio-venous O_2_ saturation difference, *S*a–v_O_2__ [ignoring the relatively small contribution of physically dissolved O_2_ in blood with average haemoglobin (Hb) concentration]. *S*a–v_O_2__ is in turn determined by the arterial and mixed venous *P*_O_2__ values (*P*a_O_2__ and *P*v_O_2__, respectively) and the shape and properties of the blood O_2_ equilibrium curve (OEC; e.g. Weber and Campbell, 2011). In fact, right-shifts of the OEC with increasing temperature or decreasing pH have classically been linked to improved rates of tissue O_2_ supply (Bohr et al., 1904; Barcroft and King, 1909). Yet, the contribution of such OEC changes to meeting increased O_2_ demands in marine ectotherms at elevated temperatures is poorly known.

Atlantic cod are of particular interest in this context because the different Hb phenotypes of their polymorphic major HbI component (Sick, 1961) have been associated with differences in the thermal sensitivity of O_2_ binding in red blood cells (RBCs) (Karpov and Novikov, 1980; Andersen et al., 2009). The frequencies of the two co-dominant alleles underpinning the HbI polymorphism vary inversely along a latitudinal cline in the north-east Atlantic, from the Barents Sea with frequencies of the HbI 1 allele as low as 0–0.1, to the southern North Sea, where HbI 1 frequency rises as high as 0.6–0.7 (Sick, 1965; Jamieson and Birley, 1989; Andersen et al., 2009; Ross et al., 2013). These clines have been attributed to natural selection acting on divergent temperature sensitivities of Atlantic cod harbouring the different HbI genotypes regarding growth, physiology and behaviour (reviewed by Andersen, 2012; Ross et al., 2013). However, the brief but influential report by Karpov and Novikov (1980) that first suggested functional differences in RBC O_2_ affinity between the HbI genotypes was based on RBC OECs of White Sea Atlantic cod (67° N) near their northern, lower thermal distribution limit and measured at a single, physiologically rather low pH value (7.5; Karpov and Novikov, 1980). Its findings and extrapolations for the efficiency of RBC O_2_ transport in Atlantic cod HbI genotypes near their southern, upper thermal limit of distribution have, to our knowledge, never been experimentally verified.

The present study was undertaken to assess the thermal sensitivity of RBC O_2_ binding, and its consequences for *S*a–v_O_2__ under *in vivo*-relevant conditions in Atlantic cod HbI genotypes near their upper thermal distribution limit in the north-east Atlantic. The results showed statistically indistinguishable RBC O_2_ affinities and pH and temperature sensitivities between all three HbI genotypes in wild-caught Atlantic cod from the Irish Sea (53° N). All animals showed an unusually low RBC O_2_ affinity, with no – or even reversed – thermal sensitivity over much of the physiological pH and temperature range. This was paired with strongly pH-dependent affinity and cooperativity of RBC O_2_ binding. Modelling of *S*a–v_O_2__ at physiological values for pH, temperature and *Ṗ*_O_2__ revealed a substantial capacity for increases in this factor to meet rising tissue O_2_ demands at 5.0 and 12.5°C, but not at 20°C, where further increases in the maximal rate of O_2_ delivery by the circulatory system are predicted to solely rely on increases in cardiac output and O_2_ capacity.

## MATERIALS AND METHODS

Wild Atlantic cod, *Gadus morhua* Linnaeus 1758, with a total length of 46.4±0.45 cm (here and elsewhere: mean±s.e.m.; *N*=106 animals) were caught by hook and line on board commercial fishing boats in the Mersey Estuary adjoining the Irish Sea near Liverpool, UK (53°25′ N, 3.02°1′ E), between mid-January and the end of February 2015 at sea surface temperatures between 6.8 and 7.9°C. Animals were killed by a British Home Office approved Schedule 1 method, involving concussion and destruction of the brain. Blood was removed from caudal vessels using heparinised 1 ml syringes, with the dead space filled with 9.000 U ml^−1^ sodium heparin solution (from porcine intestinal mucosa, Sigma-Aldrich). Up to eight animals of undetermined sex were bled on the day before each experiment and samples were kept on ice for a maximum of 10 h before landing and genotyping. Immediately after, blood of a single individual was selected for experiments the next day in accordance with a pre-determined random selection of genotype order.

### Genotype determination

RBCs were isolated from plasma and buffy coat by centrifugation (3000 rcf, 4°C, 4 min) and 20 µl of RBC pellet was lysed by adding 64 µl cold distilled water. Hbs in the haemolysate were separated by horizontal agarose gel electrophoresis, modified from Sick (1961). A 1% agar gel was prepared in diluted (1:1, with water) Smithies buffer (45 mmol l^−1^ Tris, 25 mmol l^−1^ boric acid and 1 mmol l^−1^ EDTA, adjusted to pH 8.8 at room temperature). Undiluted Smithies buffer was used as an electrode buffer and samples were run towards the positive pole at 120 V for 40 min at 4°C in a cold room, whereupon Hb bands were viewed immediately without staining.

### Preparation of RBC suspensions

The remaining RBC pellets of selected samples were resuspended in physiological saline (mmol l^−1^: NaCl 125.5, KCl 3, MgCl_2_ 1.5, CaCl_2_ 1.5, d-glucose 5 and Hepes 20, adjusted to pH 7.97 at 15°C; Koldkjaer and Berenbrink, 2007). The above washing procedure of centrifugation and resuspension in fresh saline was repeated twice and during the last step RBCs were resuspended at an approximate haematocrit (Hct) of 5–10% and stored overnight at 4°C in a 15 ml Falcon tube with a large air reservoir, placed on the side to maximise exchange surface area between saline and sedimented cells. Following the overnight rest and immediately prior to establishing RBC OECs, RBCs were washed again, resuspended in fresh saline at 8–13% Hct, and the concentrations of tetrameric Hb (Hb_4_), ATP and GTP, and mean corpuscular Hb concentration (MCHC) were determined.

### Analytical procedures

[Hb_4_] was determined by the cyan-methaemoglobin method using modified Drabkin's solution (11.9 mmol l^−1^ NaHCO_3_, 0.61 mmol l^−1^ K_3_[Fe(CN)_6_] and 0.77 mmol l^−1^ KCN) and a haem-based extinction coefficient of 11.0 l mmol^−1^ cm^−1^ at a wavelength of 540 nm, as described earlier (Völkel and Berenbrink, 2000). Hct was measured in micro-haematocrit tubes using a SpinCrit Micro-Hematocrit centrifuge and MCHC was calculated as [Hb_4_]/(Hct/100). For ATP and GTP concentration determination, equal volumes of washed RBC suspension and 0.6 mmol l^−1^ perchloric acid (PCA) were mixed before freezing at −80°C for later analysis. Samples were defrosted and centrifuged at 4°C and 13,000 rcf. The PCA extract was neutralised to an approximate pH of 7 by the addition of concentrated potassium carbonate to the supernatant and the resulting precipitate was removed by centrifugation. ATP and GTP concentrations in the supernatant were then determined enzymatically via the two-step process outlined by Albers et al. (1983), with the following modifications: the enzymes hexokinase with glucose 6-phosphate dehydrogenase (H8629, Sigma-Aldrich) and nucleoside 5′-diphosphate kinase (N0379, Sigma-Aldrich) were used at concentrations of 13 and 5000 U ml^−1^, respectively. The accuracy of the test and potential losses of nucleotide triphosphates (NTPs) during PCA extractions were examined using ATP and GTP standard solutions (A2383 and G8877, Sigma-Aldrich). Recovery was 96.4±0.9% and 80.4±0.64% (*N*=18) for ATP and GTP, respectively, and all measurements were corrected accordingly. Concentrations were converted to mmol l^−1^ RBCs using the equation presented by Albers et al. (1983), then standardised using MCHC and are presented as ATP/Hb_4_ and GTP/Hb_4_ molar ratios.

### OEC determinations

After the above measurements were taken, RBC suspensions were further diluted 10-fold in pH 7.97 saline and then pH was varied by final 10-fold dilutions in saline of pH 7.45, 7.70 and 7.97 (all adjusted at 15°C). Thermally induced saline pH changes were assessed in air-equilibrated RBC suspensions using a Lazar Model FTPH-2S pH electrode with a Jenco 6230N meter (Jenco Collaborative, CA, USA). Given the buffering properties of the saline (20 mmol l^−1^ Hepes) and small quantity of cells (0.08–0.13% Hct), oxygenation-linked changes in pH of RBC suspensions during OEC measurements were deemed negligible. For each individual, 1.2 ml aliquots of final RBC suspension were incubated, at the three pH values in parallel, in 50 ml capacity Eschweiler glass tonometers (Eschweiler GmbH, Engelsdorf, Germany) with custom-attached 1 cm path length optical glass cuvettes (following a design by Brix et al., 1998). This was performed at temperatures of 5.0, 12.5 and 20.0°C and a minimum of five *P*_O_2__ values covering the range 20–80% RBC O_2_ saturation. *P*_O_2__ was varied by mixing air and N_2_ in pre-determined ratios using a Wösthoff gas mixing pump (Wösthoff GmbH, Bochum, Germany) and the final gas mixture was fully humidified at the experimental temperature. RBC suspensions were equilibrated for at least 20 min with each gas mixture. Solutions remained sealed within the tonometer to ensure *P*_O_2__ stayed constant while an optical spectrum was taken between 500 and 700 nm (Unicam UV 500 spectrophotometer, Thermo Electron Corporation, OH, USA; with Vision 32 software) and O_2_ saturation of RBC suspensions was determined by spectral deconvolution (Völkel and Berenbrink, 2000).

### Data analysis and statistics

Spectral deconvolution of the optical spectra (see Völkel and Berenbrink, 2000) was used to determine the concentrations of Hb derivatives within RBC suspensions (oxyhaemoglobin, HbO_2_; deoxyhaemoglobin, deoxyHb; and the two forms of methaemoglobin, acid Hb^+^ and alkaline Hb^+^) at each temperature, pH and *P*_O_2__ value using SigmaPlot 12.5 (Systat Software Inc., San Jose, CA, USA). The unknown concentrations (mmol l^−1^) of the different tetrameric Hb derivatives were calculated using:
(2)
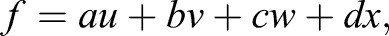


where *a*, *b*, *c* and *d* represent [HbO_2_], [deoxyHb], [acid Hb^+^] and [alkaline Hb^+^], respectively, and were restricted to values greater than or equal to zero; *f* is the predicted dependent variable to be fitted to the measured absorption data for each nm step between 500 and 700 nm; and *u*, *v*, *w* and *x* represent the respective experimentally determined absorption coefficients for each Hb derivative at each wavelength between 500 and 700 nm, respectively. Absorption coefficients for HbO_2_ and deoxyHb were created with RBC suspensions in pH 8.05 saline at 5.0°C, exposed to 100% oxygen or 100% nitrogen. Acid Hb^+^ and alkaline Hb^+^ absorption coefficients were constructed using Hb suspensions oxidised with tri-potassium hexacyanoferrat at pH 6.5 and 8.05, respectively, although the analysis showed that no methaemoglobin formation had occurred in any of our samples. In all cases, the predicted values by the curve-fitting procedure were plotted for each wavelength between 500 and 700 nm together with the measured spectra for visual inspection of the accuracy of the prediction.

The level of RBC O_2_ saturation (*S*) was calculated as [HbO_2_]/([HbO_2_]+[deoxyHb]). Hill plots on data between 20% and 80% saturation were created using log[*S*/(1−*S*)] versus log*P*_O_2__. log*P*_50_ was calculated by linear regression as the log*P*_O_2__ when log[*S*/(1−*S*)] equalled 0. The slope of the regression line indicated the apparent cooperativity of RBC O_2_ binding or Hill number (*n*_H_). The Bohr coefficient was calculated by Φ=Δlog*P*_50_/ΔpH for each pH interval. Because of non-linearity, at each temperature, log*P*_50_ and *n*_H_ were plotted against measured saline pH and 2nd order polynomials were used to standardise them to pH 7.40, 7.65 and 7.9, removing the effect of temperature-induced pH shifts on these variables. Once standardised to fixed pH, thermal sensitivities of OECs were expressed as apparent heat of oxygenation, Δ*H*′. These were calculated using the van't Hoff equation Δ*H*′=2.303*R*[Δlog*P*_50_/(Δ1/*T*)], where *R* is the universal gas constant (0.008314 kJ K^−1^ mol^−1^) and *T* is temperature in K.

OECs for a series of fixed pH values were produced using values for *n*_H_ and *P*_50_ predicted at a given pH for each individual from the same 2nd order polynomial equations used above for standardising log*P*_50_ and *n*_H_. RBC O_2_ saturation *S* was then calculated as a function of *P*_O_2__ using:
(3)
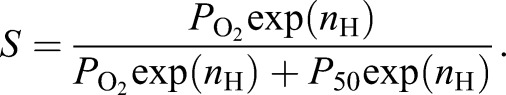


*S*a–v_O_2__ during acute temperature and/or pH changes was modelled as the difference between *S*a_O_2__ and *S*v_O_2__ at physiologically relevant pH and arterial and venous *P*_O_2__ values read from RBC OECs. An arterial pH of 7.86 and average values of 85 and 30 mmHg for *P*a_O_2__ and *P*v_O_2__ were assumed for resting normoxic Atlantic cod at 12.5°C, based on literature values for this species close to this temperature (Kinkead et al., 1991; Perry et al., 1991; Claireaux and Dutil, 1992; Nelson et al., 1996; Larsen et al., 1997; Karlsson et al., 2011; Petersen and Gamperl, 2011). *P*a_O_2__ was assumed constant during acute thermal change (Sartoris et al., 2003), whereas values for *P*v_O_2__ at 5.0 and 20.0°C of 60 and 15 mmHg, respectively, were based on the percentage changes observed by Lannig et al. (2004). Changes in arterial pH were assumed to follow the relationship with temperature established for marine teleosts and elasmobranchs by Ultsch and Jackson (1996). Owing to the generally larger deoxygenation-linked proton uptake in teleost Hbs compared with those of other vertebrates (Berenbrink et al., 2005), venous pH was assumed to be similar to arterial pH, as previously recorded in normoxic Atlantic cod (Perry et al., 1991).

Maximal *S*a–v_O_2__ at each temperature was taken as the maximally observed *S*a–v_O_2__ at any pH and *P*a_O_2__ and *P*v_O_2__ equalling 85 and 15 mmHg, the lowest average *P*v_O_2__ reported for Atlantic cod in the literature under any condition.

All values are reported as means±s.e.m. Sigmaplot 12.5 (Systat Software Inc.) was used for all statistical analysis and significance was accepted at *P*<0.05. Differences between mean values were generally assessed by one-way ANOVA, followed by a *post hoc* Tukey test, if relevant. Other test statistics (two- and three-way ANOVA, χ^2^ and one-sample *t*-tests) were used as indicated in the text.

## RESULTS

In 106 Atlantic cod caught between mid-January and the end of February 2015 in the River Mersey Estuary near Liverpool, UK, the HbI 1/1 genotype dominated (45% of individuals), followed by 41% HbI 1/2 heterozygotes and just 14% HbI 2/2 homozygotes (Table 1). These genotype frequencies did not significantly deviate from the expectations according to the Hardy–Weinberg equilibrium (χ^2^=1.09, d.f.=2, *P*>0.5) or from the averaged values recorded for the Irish Sea between 1971 and 1977 (χ^2^=5.73, d.f.=2, *P*>0.05; Jamieson and Birley, 1989). HbI 1 allele frequency was 0.66 and thus among the highest values recorded for Atlantic cod stocks across their geographical range (Ross et al., 2013), and similar to values reported in recent years for the southern North Sea (0.66, Pörtner et al., 2001; 0.64, Andersen et al., 2009). There was no difference in total length between HbI genotypes in 84 animals that were available for length measurement, or in the subset of 16 animals selected for OECs (*P*=0.073 and 0.226, respectively; Table 1). In the latter group, there were also no significant HbI genotype-related differences in Hct (*P*=0.834), Hb concentration (*P*=0.697), MCHC (*P*=0.371) and ATP/Hb_4_ (*P*=0.284) or GTP/Hb_4_ (*P*=0.620) ratios of washed RBC suspensions immediately prior to experiments (Table 1). Furthermore, the ATP/Hb_4_ and GTP/Hb_4_ ratios were similar to values previously reported for whole blood (Leray, 1982).

**Table 1.**
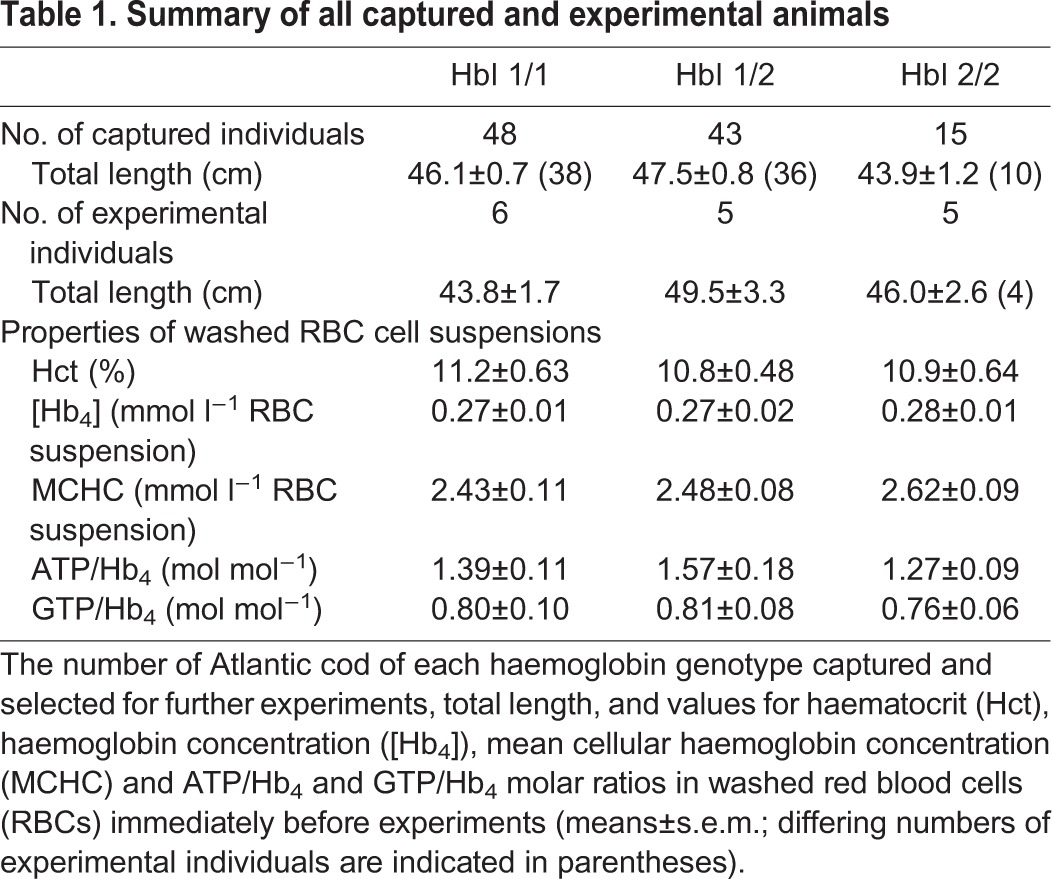
**Summary of all captured and experimental animals**

**Fig. 1.**
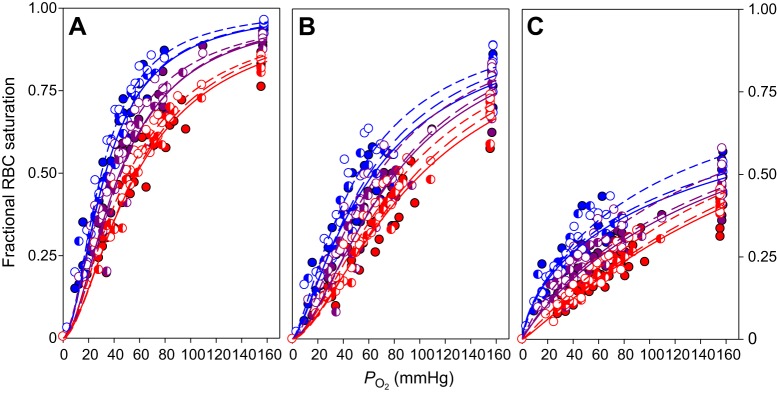
**Oxygen equilibrium curves of Atlantic cod red blood cells (RBCs) with different haemoglobin HbI genotypes.** Data are for 5.0, 12.5 and 20.0°C (blue, purple and red, respectively) and at nominal saline pH values of (A) 7.90, (B) 7.65 and (C) 7.40. Circles indicate measured values while lines are based on sigmoidal curve fits for each temperature and HbI genotype (HbI 1/1, solid lines, filled symbols, *N*=6; HbI 1/2, long-dashed lines, half-filled symbols, *N*=5; HbI 2/2, short-dashed lines, open symbols, *N*=5). For each individual, five data points were obtained at each pH and temperature.

OECs of Atlantic cod RBCs at all three temperatures and for all three HbI genotypes revealed strong Bohr and Root effects, as shown by strong pH-induced reductions in RBC O_2_ affinity and O_2_ saturation at atmospheric *P*_O_2__, respectively (Fig. 1). At each nominal saline pH, increasing temperature appeared to reduce O_2_ affinity, shifting OECs to the right and increasing *P*_50_ (Fig. 1). However, this effect will have been partially due to the temperature-induced shifts in the pH of the Hepes buffer. Thus, for example, the actual pH values experienced by RBCs suspended in saline with a nominal pH of 7.90 were 7.99, 7.89 and 7.81 at 5.0, 12.5 and 20.0°C, respectively, with s.e.m. values for pH below 0.005.

**Fig. 2.**
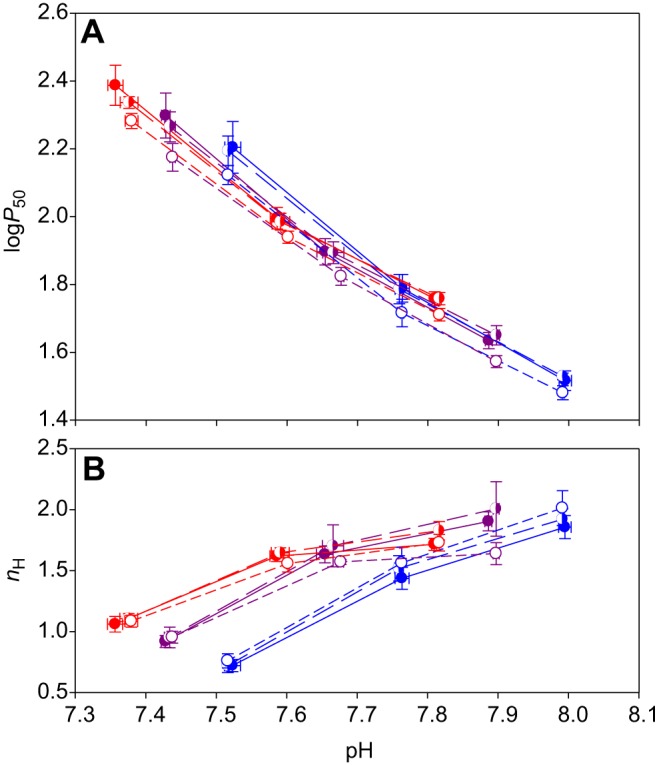
**Effect of pH, HbI genotype and temperature on the affinity and cooperativity of O_2_ binding in Atlantic cod RBCs.** (A) Mean±s.e.m. log*P*_50_ versus pH for HbI 1/1 (filled symbols, solid lines, *N*=6), HbI 1/2 (half-filled symbols, long-dashed lines, *N*=5) and HbI 2/2 (open symbols, short-dashed lines, *N*=5), at 5.0, 12.5 and 20.0°C (blue, purple and red, respectively). *P*_50_ was measured in mmHg. (B) Mean±s.e.m. *n*_H_, Hill's cooperativity coefficient, at 50% RBC O_2_ saturation, for the same data as in A.

In the Bohr plot (Fig. 2A), the stepwise reduction of pH from nominal pH 7.90 to 7.65 and then 7.40 resulted in significant increases in log*P*_50_ within all genotypes and all temperatures (*P*<0.001). Thus, the southern HbI 1/1 genotype at 5.0°C and pH 7.99 had a log*P*_50_ of 1.52±0.02 (corresponding to a *P*_50_ of 33 mmHg). As pH decreased, O_2_ affinity showed a corresponding decrease, with a log*P*_50_ of 1.79±0.03 (*P*_50_ of 62 mmHg) at pH 7.75 and a further decrease at pH 7.51 to 2.20±0.06 (*P*_50_ of 158 mmHg). Similar effects of pH were also observed at 12.5 and 20.0°C, although increasing temperatures caused a general shift of curves towards higher log*P*_50_ values and lower pH values (Fig. 2).

Surprisingly, log*P*_50_ values were not affected by HbI genotype at any tested pH or temperature (*P*=0.161–0.421), although there was a tendency for values in the northern HbI 2/2 type to be consistently lower than those of the other two genotypes.

The relationship between log*P*_50_ and pH appeared distinctly curvilinear and a three-way ANOVA with pH range, temperature and genotype as factors revealed that the Bohr coefficient φ, Δlog*P*_50_/ΔpH, significantly increased in magnitude from around −1.08 in the higher pH range to about −1.65 in the lower pH range (*P*<0.001). This increased pH dependence of RBC O_2_ affinity at lower pH is likely to be due to the more pronounced Root effect at the lowest pH values. Both genotype and temperature had no significant effect on the Bohr coefficient (*P*=0.183 and 0.840, respectively).

Hill's cooperativity constant *n*_H_ did not vary significantly between the upper two saline pH values at any temperature, attaining values between 1.5 and 2.0 (Fig. 2B). At the lowest saline pH, however, *n*_H_ was significantly reduced down to values between 1.0 and 0.7 compared with the highest saline pH (*P*<0.001), indicating the onset of the Root effect. Similar to log*P*_50_ above, *n*_H_ also remained unaffected by HbI genotype at all pH values and temperatures (*P*=0.161–0.421). Given the lack of significant Hb genotype differences in all analyses above, data for all animals were pooled for the following analyses.

After standardising log*P*_50_ values of the combined HbI genotypes to constant pH values (Table 2), log *P*_50_ at pH 7.65 was completely independent of temperature over the entire range from 5.0 to 20.0°C (Table 3). At pH 7.90, log *P*_50_ was also statistically indistinguishable between 5.0°C and 12.5°C, and only increased significantly at 20.0°C compared to these values (*P*=0.002 and *P*<0.001, respectively; Table 3). At pH 7.40, log*P*_50_ was unaffected by temperature between 20.0 and 12.5°C, and only significantly increased at 5°C compared to these values (*P*<0.001), revealing a reversed temperature sensitivity at the lower temperature range.

**Table 2.**
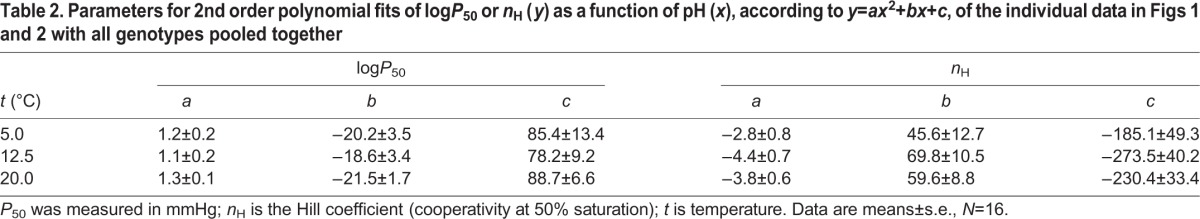
**Parameters for 2nd order polynomial fits of log*P*_50_ or *n*_H_ (*y*) as a function of pH (*x*), according to *y*=*ax*^2^+*bx*+*c*, of the individual data in Figs 1 and 2 with all genotypes pooled together**

**Table 3.**
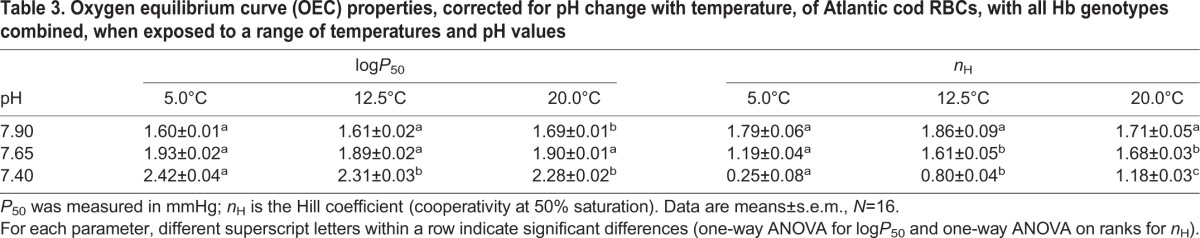
**Oxygen equilibrium curve (OEC) properties, corrected for pH change with temperature, of Atlantic cod RBCs, with all Hb genotypes combined, when exposed to a range of temperatures and pH values**

The pH-adjusted cooperativity coefficient *n*_H_ (Table 2) was unaffected by temperature at pH 7.9 (*P*=0.412; Table 3), but at pH 7.65 it was significantly reduced at 5.0°C when compared with that at 12.5 and 20.0°C (*P*<0.001), although values at 12.5 and 20°C did not differ significantly. At pH 7.4, *n*_H_ significantly increased with temperature over the whole range (*P*<0.001; Table 3).

**Fig. 3.**
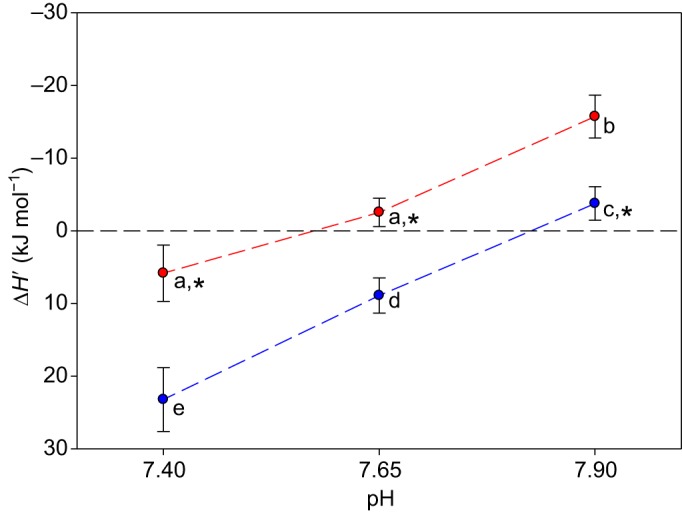
**Apparent heat** **of oxygenation, Δ*H*′, for Atlantic cod RBCs.** Values between 5.0 and 12.5°C (blue) and 12.5 and 20.0°C (red) are shown at each reference pH (means±s.e.m., *N*=16). Note reversal of the *y*-axis, with negative values denoting an exothermic reaction at the top. Different letters within a temperature interval or at constant pH indicate significantly different Δ*H*′ values (two-way ANOVA). *Values not significantly different from zero (one-sample *t*-test).

Δ*H*′ for the oxygenation reaction of Atlantic cod RBCs was significantly affected by both pH (*P*<0.001) and temperature range (*P*<0.001), with no significant interaction (*P*=0.574) between factors (two-way ANOVA, with temperature range and pH as factors; Fig. 3). Between 12.5 and 20.0°C and at pH 7.90, Atlantic cod RBCs showed a typical exothermic oxygenation reaction, with a negative Δ*H*′ value of −15.7±2.9 kJ mol^−1^. However, in the same thermal range, thermal sensitivity was significantly reduced at pH 7.65 and 7.40, where Δ*H*′ values amounted to −2.5±1.9 and +5.8±3.9 kJ mol^−1^, respectively. These values were not significantly different from each other and one-sample *t*-tests showed that they also did not significantly differ from zero (*P*=0.208 and 0.158, respectively; Fig. 3). At all pH values, the magnitude of Δ*H*′ was significantly higher between 5.0 and 12.5°C than between 12.5 and 20.0°C. In the lower temperature range at pH 7.9, this resulted in a Δ*H*′ value of −3.8±2.3 kJ mol^−1^, which was not significantly different from zero (one-sample *t*-test, *P*=0.119). Stepwise, significantly more endothermic RBC oxygenation was observed at pH 7.65 (+8.9±2.4 kJ mol^−1^) and then pH 7.40 (+23.2±4.4 kJ mol^−1^).

**Fig. 4.**
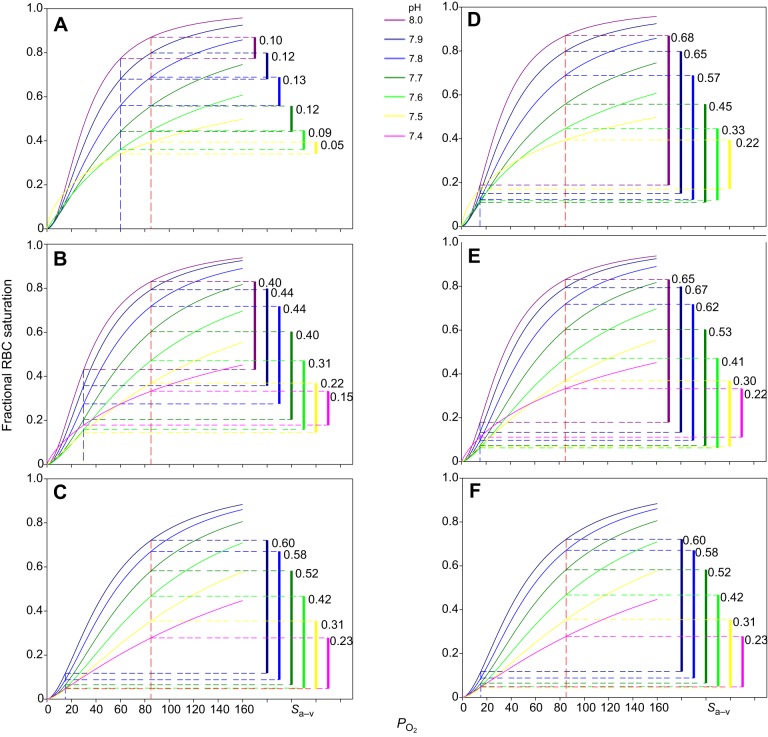
**Modelled RBC O_2_ equilibrium curves (OECs) and arterio-venous O_2_ saturation differences in Atlantic cod at different values for pH and temperature.** OECs are shown for a series of standardised pH values and temperatures of 5.0°C (A,D), 12.5°C (B,E) and 20.0°C (C,F). Red dashed vertical lines indicate routine arterial *P*_O_2__ values, *P*a_O_2__. Blue dashed vertical lines indicate either resting mixed venous *P*_O_2__ values (*P*v_O_2__, A–C) or minimally observed mixed venous *P*_O_2__ values (*P*v_O_2__­_,min_, D–F) (see Materials and methods). Corresponding arterial and venous O_2_ saturation, *S*a_O_2__ and *S*v_O_2__, and their difference, *S*a–v_O_2__, are indicated for each pH by colour-matched horizontal dashed lines and vertical bars, respectively. Because of pH shifts with temperature in the underlying data set (Fig. 2), OECs were not modelled for pH 8.0 at 20.0°C and pH 7.4 at 5.0°C.

Using 2nd order polynomials (Table 2), log*P*_50_ and *n*_H_ values from Fig. 2 were standardised for a series of fixed pH values and the corresponding OECs shown for three temperatures (Fig. 4). At each temperature, literature values for *in vivo P*a_O_2__ and *P*v_O_2__ and the resulting *S*a–v_O_2__ are indicated for each pH. The curves suggest *in vivo* arterial O_2_ saturations, across temperature, at resting arterial pH (7.91–7.81 between 5.0 and 20.0°C, respectively) and constant arterial *P*_O_2__ (85 mmHg) of no more than 80% (Fig. 4A–C). Increasing temperatures are associated with greater use of the venous reserve, as shown by decreases in *P*v_O_2__, and consequent increases in *S*a–v_O_2__ from 0.11 at 5.0°C to 0.44 and 0.58 at 12.5°C and 20.0°C, respectively. Further, at each temperature and with fixed *P*a_O_2__ and *P*v_O_2__ values, acidification-induced decreases in *S*v_O_2__ were accompanied by similar, or even greater decreases in *S*a_O_2__ (Fig. 4A–C). This suggests that in Atlantic cod RBCs the benefits of the Bohr effect under general acidosis in facilitating O_2_ offloading to tissues at a given *P*v_O_2__ are minimised by parallel or even greater decreases in arterial O_2_ loading.

Estimates of maximal *S*a–v_O_2__ values at 5, 12.5 and 20.0°C (Fig. 4D–F) show a substantial potential for increasing *S*a–v_O_2__ above routine values at 5.0 and 12.5°C, where *S*a–v_O_2__ rises by factors of 4–5 and 1.5–2.0, respectively, when *P*v_O_2__ is allowed to drop to the minimally observed value of 15 mmHg (Fig. 4E,F versus A,B). However, there was no additional capacity for *S*a–v_O_2__ increases above routine values at 20.0°C (Fig. 4C,F). Similarly, across pH values, maximal *S*a–v_O_2__ values tended to decrease, rather than increase, with temperature, such that even taking into account a temperature-associated decrease in *in vivo* arterial pH from 7.91 at 5.0°C to 7.81 at 20.0°C did not increase *S*a–v_O_2___ _(Fig. 4D–F).

## DISCUSSION

The results of the present study suggest that the O_2_ binding properties of Atlantic cod RBCs near their southern, upper thermal distribution limit in the north-east Atlantic are, contrary to common expectations, independent of HbI genotype, characterised by an unusually low O_2_ affinity that is strongly affected by pH and remarkably temperature insensitive over much of the physiological pH range. These factors combine to create a blood O_2_ transport system in which maximal *S*a–v_O_2__ under *in vivo* conditions does not increase with temperature or general blood acidosis, which universally accompanies elevated temperature across ectothermic vertebrates (Ultsch and Jackson, 1996). This is surprising in light of the fact that increased temperature and general blood acidification are the classic textbook examples of how the rate of O_2_ supply to tissues can be increased by right-shifts of the OEC and increased *S*a–v_O_2__ (Barcroft and King, 1909; Bohr et al*.,* 1904; Dejours, 1975; Berenbrink, 2006, 2011a). Similarly, temperature-dependent differences in O_2_ affinity between the HbI genotypes of Atlantic cod were thought to be crucial in the adaptation of this species to environmental temperature for more than 35 years (Karpov and Novikov, 1980; Andersen, 2012; Ross et al., 2013). The clear lack of both a temperature and HbI genotype effect on RBC O_2_ affinity demonstrated in the present study, together with results from carefully controlled whole-animal studies (Gamperl et al., 2009), points to an emerging paradigm shift in our understanding of thermal adaptation of O_2_ supply mechanisms and the roles of HbI genotype differences in Atlantic cod. In the following discussion, the results are critically evaluated and the underlying mechanisms and consequences for maximal circulatory O_2_ supply rates of Atlantic cod at elevated temperatures are discussed.

### Low O_2_ binding affinity of Atlantic cod RBCs

The average *P*_50_ of Atlantic cod RBCs across the three genotypes was 40 mmHg (calculated from log*P*_50_ values at pH 7.90 between 5.0 and 12.5°C in Table 3). This value is among the lowest O_2_ affinities that have been reported for blood or RBCs of any fish under the standardised conditions given above (e.g. Herbert et al., 2006). Such a low *P*_50_ results in arterial blood in gills lying on the edge of the steep part of the OEC, with modelled RBC O_2_ saturations of no more than 80% at typical *P*_O_2__ and pH values and at any temperature between 5.0 and 20.0°C (Fig. 4). This guarantees that across all temperatures, small decreases in venous *P*_O_2__ enable large increases in O_2_ unloading in the tissues at a relatively high venous *P*_O_2__, which will safeguard a sufficiently large diffusion gradient from the blood plasma to tissue mitochondria. Blood O_2_ tissue extraction [*S*a–v_O_2__/*S*a_O_2__] was accordingly as high as 53% for normoxic resting animals at pH 7.90 and 12.5°C (calculated from Fig. 4A), which compares well with estimates in Atlantic cod *in vivo* under similar conditions (57%, Perry et al., 1991; 51%, Petersen and Gamperl, 2011). The high venous unloading *P*_O_2__ may be particularly important for cardiac O_2_ supply in species like Atlantic cod, where the ventricle lacks a coronary blood supply and consists entirely of spongey myocardium that relies exclusively on the O_2_ remaining in luminal blood that is returned from the other tissues (Santer and Walker, 1980; Farrell et al., 2012). However, too low a blood O_2_ affinity comes at the cost of potentially reducing the efficiency of a further right-shift of the OEC for increasing *S*a–v_O_2__ under, for example, warming or general acidosis.

### (In)efficiency of the Bohr effect in enhancing O_2_ supply under general acidosis

The low O_2_ affinity of Atlantic cod RBCs was paired with one of the largest Bohr effects reported for blood or RBCs under controlled standard conditions (Δlog*P*_50_/ΔpH=−1.08±0.05, pH 7.9 to 7.65 and 5.0 to 20.0°C). At still lower pH values, the pH-induced decline in RBC O_2_ affinity was associated with a reduced cooperativity of RBC O_2_ binding and with O_2_ saturations below 60% in air-equilibrated RBCs. This indicated a strong Root effect and confirmed the positive correlation between the magnitudes of the Bohr and Root effects that has been found across a wide range of diverse ray-finned fishes (Berenbrink et al., 2005). Low O_2_ affinity and a strong Bohr effect were both previously reported for Atlantic cod haemolysates in the presence of saturating ATP concentrations (Pörtner et al., 2001; Brix et al., 2004; Verde et al., 2006). Importantly, these findings on Hb solutions in artificial buffers also closely reflect results for Atlantic cod whole blood in the presence of a physiological CO_2_/bicarbonate buffer system (Herbert et al., 2006). Bohr et al. (1904) first emphasised the biological importance of elevated blood carbon dioxide partial pressure (*P*_CO_2__) and thereby blood acidification for enhancing blood O_2_ utilisation in the tissues, without affecting O_2_ uptake at the higher *P*_O_2__ values in the respiratory organ. The present study surprisingly suggests that these generally accepted benefits of the Bohr effect are partially cancelled in Atlantic cod as a result of their low blood O_2_ affinity, whereby any decrease in *S*v_O_2__ during general acidosis is accompanied by a similar or even larger decrease in *S*a_O_2__, such that *S*a–v_O_2__ remains the same or even decreases upon acidification (Fig. 4). Thus, the unusually large effect of elevated CO_2_ or low pH on Atlantic cod RBC O_2_ binding affinity and capacity (Krogh and Leitch, 1919; Herbert et al., 2006; Berenbrink et al., 2011) will be mainly useful during localised tissue acidification, such as at the tissue poles of the vascular counter-current exchangers (*retia mirabilia*) in the eye and swim bladder of Atlantic cod, where they are crucial for generating super-atmospheric *P*_O_2__ values that support the high metabolic demands of the poorly vascularised retina, and for swim bladder gas filling against increasing hydrostatic pressures at depth (Bohr, 1894; Wittenberg and Wittenberg, 1962; Berenbrink et al*.*, 2005; Berenbrink, 2007).

These considerations do not negate the benefits of the Bohr effect in increasing *S*a–v_O_2__ because of arterio-venous pH differences that are caused by the differences in arterial and venous *P*_CO_2__ or by selective short-circuiting of catecholamine-activated RBC intracellular pH regulation in tissues with plasma-accessible carbonic anhydrase, as recently suggested for rainbow trout (Rummer et al., 2013). Instead, they emphasise that parallel pH shifts in arterial and venous blood, such as during exercise-induced lactacidosis or environmental warming, are unlikely to increase *S*a–v_O_2__ in Atlantic cod at physiological *P*a_O_2__ and minimal *P*v_O_2__. Any increases in circulatory blood O_2_ supply under these conditions must come from increases in cardiac output, blood O_2_ capacity or alternative mechanisms that may increase *S*a–v_O_2__.

### Reduced and reversed thermal sensitivity of O_2_ binding in Atlantic cod RBCs

Whole-body or local increases in temperature, such as in working muscle, are classically thought to increase blood O_2_ transport by increasing *S*a–v_O_2__ (Barcroft and King, 1909). In many animals, the intrinsically exothermic nature of haem O_2_ binding determines the overall heat of Hb oxygenation, resulting in a lowered Hb O_2_ affinity at elevated temperature (Weber and Campbell, 2011). However, binding of allosteric effectors such as protons and ATP or GTP preferentially to deoxyHb requires their endothermic release during oxygenation and this can compensate for the heat released by exothermic haem oxygenation, leading to a reduced or even reversed temperature sensitivity of Hb O_2_ affinity. This is best known for heterothermic tuna, billfishes and lamnid sharks, where exothermic Hb O_2_ binding may cause problems in heat-conserving vascular counter-current exchangers (Weber and Campbell, 2011). The finding of largely thermally insensitive RBC O_2_ affinity in Atlantic cod in this study, together with the study by Clark et al*.* (2010) on Pacific mackerel, suggests that low thermal sensitivity of RBC O_2_ affinity may be more widespread among ectotherm fishes than previously thought.

Normally, with an overall exothermic reaction of Hb O_2_ binding, increased temperatures decrease Hb O_2_ affinity and cause a right-shift of the OEC. This will generally allow an increased *S*a–v_O_2__ in any organism with *S*a_O_2__ and *P*a_O_2__ in the flat upper part of the OEC because a decrease in *S*v_O_2__ allows a greater exploitation of the venous reserve. However, for a species with a RBC O_2_ affinity as low as reported for Atlantic cod in the present study, any gain in O_2_ offloading by a decrease in *S*v_O_2__ will be obliterated by a parallel decrease in *S*a_O_2__ at typical *P*a_O_2__. This may be the ultimate, evolutionary driving cause of the reduced thermal sensitivity of O_2_ binding in Atlantic cod RBCs.

The proximate, mechanistic explanation for the phenomenon may involve at least two not necessarily exclusive factors. First, the large Bohr effect suggests an above average increase in the number of proton binding sites in deoxyHb compared with oxyHb (for review, see Berenbrink, 2006, 2011a). The release of these protons during oxygenation may compensate for exothermic haem O_2_ binding. This is supported by the strong effect of pH on the overall enthalpy of RBC oxygenation over the whole temperature range (Fig. 3). Second, the increase in cooperativity of RBC O_2_ binding with temperature at low pH (Table 3) suggests that the over-stabilisation of deoxyHb by the Root effect (with *n*_H_≤1; see Berenbrink, 2011b) is weakened at higher temperatures, where increasing values of *n*_H_ indicate an endothermic transition to the oxy conformation of Hb. This is consistent with previous work demonstrating the large endothermic nature of the deoxyHb to oxyHb conformational transition in teleosts (Saffran and Gibson, 1979). In addition, the endothermic release of the organic phosphate modulators ATP and GTP from deoxyHb upon oxygenation may contribute to the overall heat of oxygenation of Atlantic cod RBCs, a mechanism that has previously been shown to contribute to the reduced and reversed oxygenation enthalpy of several species of billfish (Weber et al., 2010). However, elucidation of the detailed molecular mechanism(s) behind reduced or even reversed thermal sensitivity of Atlantic cod RBC O_2_ affinity awaits detailed studies on purified Hbs under tightly controlled conditions of allosteric modifiers.

### Lack of HbI genotype effects on O_2_ binding in Atlantic cod RBCs

The increased frequency of the HbI 1 allele towards the southern range of Atlantic cod has been widely related to a parallel cline in environmental temperature and to a presumed advantage of HbI 1/1 cod in having a higher RBC O_2_ affinity at temperatures above 15°C compared with HbI 2/2 cod where this is higher below 15°C (e.g. Karpov and Novikov, 1980; Andersen et al., 2009; reviewed by Andersen, 2012, and Ross et al., 2013). The current study establishes the absence of any statistically supported differences in the RBC O_2_ binding characteristics between Atlantic cod of all three HbI genotypes near their southern upper thermal distribution limit. This result has been consistently obtained over a range of pH values at each of three physiologically relevant temperatures and is considered robust, because factors well known to modify the genetically determined, intrinsic O_2_ binding affinity of Hb inside RBCs have been carefully controlled. To ensure environmental relevance but at the same time minimise differences in prior thermal or hypoxic acclimatisation of individuals, RBCs were obtained immediately after capture from wild Atlantic cod at a single location and over a 6 week period in winter where long-term annual water temperature changes were minimal and stratification was absent (Neat et al., 2014; O'Boyle and Nolan, 2010). In contrast to earlier studies (Karpov and Novikov, 1980; Gollock et al., 2006; Petersen and Gamperl, 2011), RBCs were washed in glucose-containing physiological saline and incubated overnight before experimentation. This removes any catecholamine hormones, which are known to be released into plasma during blood sampling stress and modify the concentration of intracellular allosteric modifiers of Hb O_2_ binding, and allows any catecholamine-initiated effects to wear off during pre-incubation in standardised physiological saline (Berenbrink and Bridges, 1994a,b). This ensures equilibration of extracellular and intracellular ion concentrations and well-defined RBC extracellular and intracellular pH values (Berenbrink and Bridges, 1994a,b) and resulted in comparable RBC intracellular Hb and nucleotide triphosphate concentrations between HbI genotypes that were similar to values in fresh whole blood (Table 1; Leray, 1982). Extreme dilution of RBCs (Hct 0.08–0.13%) in buffered physiological saline ensured full control of RBC extracellular pH and ion composition during the actual OEC measurements and avoided the need for correction of points on the OECs to constant pH, which may otherwise vary by more than 0.1 pH units with oxygenation status in Atlantic cod whole blood *in vitro* (Herbert et al., 2006). Extreme dilution also avoided potential problems with RBC O_2_ consumption that may have been behind a zero O_2_ content at *P*_O_2__ values of 15 mmHg in OECs obtained at high Hct with a gasometric method (Gollock et al., 2006; Petersen and Gamperl, 2011). Full spectrophotometric assessment of RBC O_2_ saturation between 500 and 700 nm in the present study also avoided having to assume full RBC O_2_ saturation at some arbitrary high *P*_O_2__ which may have led to a systematic overestimation of O_2_ saturation and affinity in some previous studies (Karpov and Novikov, 1980; Gollock et al., 2006; Herbert et al., 2006; Petersen and Gamperl, 2011). Finally, 5–6 specimens per HbI genotype were used to reduce outlier effects in the interpretation of the results. Together, this makes the present study the most comprehensive test yet for HbI genotype differences in RBC O_2_ binding properties. The negative finding in this study raises the question: what other characteristic(s), if any, of the different HbI alleles is behind the documented differences in geographical distribution, growth rates, hypoxia tolerance and preference temperature (reviewed by Andersen, 2012; Ross et al., 2013)?

### Possible reasons for the variability of HbI genotype effects

In theory, any potentially existing genetic differences in the intrinsic O_2_ binding characteristics between the Hb genotypes, or in their interactions with allosteric modulators such as organic phosphates, could have been masked in the present study by the large phenotypic plasticity in Hb O_2_ binding properties of ectotherms (Weber and Jensen, 1988). However, despite several attempts, the alleged large genotype effects reported for RBCs by Karpov and Novikov (1980) have been difficult to reproduce in haemolysates of the different genotypes in the presence of controlled levels of allosteric modifiers (e.g. in both the presence and the absence of ATP; Brix et al., 1998; Colosimo et al., 2003; Brix et al*.*, 2004). This rather suggests that the differences found by Karpov and Novikov (1980) at the RBC level may have been due to phenotypic plasticity rather than Hb genotype, such as different levels of intracellular organic phosphates or different degrees of catecholamine stimulation. Unfortunately, we do not have any information on RBC organic phosphate levels or treatments aimed at controlling catecholamine effects from Karpov and Novikov's (1980) study. Thus, while there is evidence for effects of Hb genotype on Atlantic cod behaviour in thermal choice experiments (Petersen and Steffensen, 2003; Behrens et al., 2012), the present study shows that they are not necessarily due to differences in RBC oxygen affinity. These considerations are in line with Gamperl et al*.* (2009), who have suggested that the adaptive value of the different Atlantic cod Hb genotypes on O_2_ supply rates in different environments may have been overemphasised.

As an alternative explanation, natural selection of HbI genotypes may act on life history stages other than the juveniles or adults that are most commonly studied. For example, unfertilised eggs of Atlantic cod have been shown to contain transcripts of all four major adult expressed globins, including the β1 globin responsible for the HbI polymorphism (Wetten et al., 2010). The functional relevance of these gene products, by necessity of maternal origin, is unclear and transcripts disappear upon fertilisation in the embryonic stages until expression is switched on again later in juveniles and adults (Wetten et al., 2010). However, if the maternal HbI genotype in eggs affects their fertilisation success, then this may explain the significantly skewed HbI genotype ratios in offspring of heterozygote parents that was observed by Gamperl et al. (2009) and was later in life balanced by significantly higher growth rates of the under-represented genotype. Thus, differing costs and benefits during different life history stages and/or in different micro-environments may lead to balanced HbI polymorphisms that differ in HbI 1 frequency across the distribution range.

In addition, the HbI polymorphism may be genetically linked to other traits that are under selection, such as the regulatory polymorphism of the HbI promoter in Atlantic cod (Star et al., 2011; Andersen, 2012), which may be responsible for the HbI genotype-associated differences in Hct and Hb concentration observed in some studies (Mork and Sundnes, 1984). Clearly, we are only just beginning to understand the molecular mechanisms enabling adaptation of marine ectotherms to environmental temperature change and more studies linking the genetics, physiology, ecology and evolution of these organisms are required.

### Concluding remarks on physiological consequence of Atlantic cod RBC O_2_ binding characteristics

Atlantic cod are regularly exposed to acute temperature shifts in their natural environments, similar to those employed in the present study; for example, during upwelling and turbulent mixing events of water bodies with different temperatures (Freitas et al*.*, 2015), or when crossing the thermocline (Righton et al*.*, 2010). The latter is particularly relevant for Irish Sea cod that continue actively changing depth during the warmer summer months, compared with North Sea cod that remain confined in bottom waters from June to September (Righton et al., 2001; Righton and Metcalfe, 2002). Our modelling approach suggests that during acute warming the O_2_ binding characteristics of Atlantic cod RBCs will enable uncompromised gill O_2_ loading at *in vivo* arterial *P*_O_2__ values and at the same time permit increased O_2_ offloading at falling venous *P*_O_2__. However, the theoretical maximal *S*a–v_O_2__ at physiological pH and arterial and venous *P*_O_2__ does not increase with temperature (Fig. 4D–F), and is already reached under conditions of acute gradual warming to 20°C (Fig. 4C,F). Under these conditions, Atlantic cod can only further increase the capacity of their circulatory O_2_ transport system by increasing blood O_2_ capacity and/or cardiac output. However, in a complex network of feedback systems, an increase in cardiac output may itself be limited, firstly by low *P*_O_2__ of cardiac luminal blood returning from systemic tissues, secondly by an increased cardiac workload and thus O_2_ demand imposed by the higher viscosity of blood with an increased RBC number, and lastly by O_2_ supply-independent physiological and anatomical limits to cardiac performance such as maximal heart rate and ventricle size, respectively. Ultimately, when all these avenues to increase blood O_2_ transport rate are exhausted, long-term preservation of aerobic scope for activity at elevated temperature may rely on the extent to which standard metabolic rate can be reduced by thermal acclimatisation.
